# Supplementary figures and images for: Contact zone of slow worms Anguis fragilis Linnaeus, 1758 and Anguis colchica (Nordmann, 1840) in Poland
Source: PeerJ. 2025 Jan 6;13:e18563. doi: 10.7717/peerj.18563 (PMC11716018; doi:10.7717/peerj.18563)

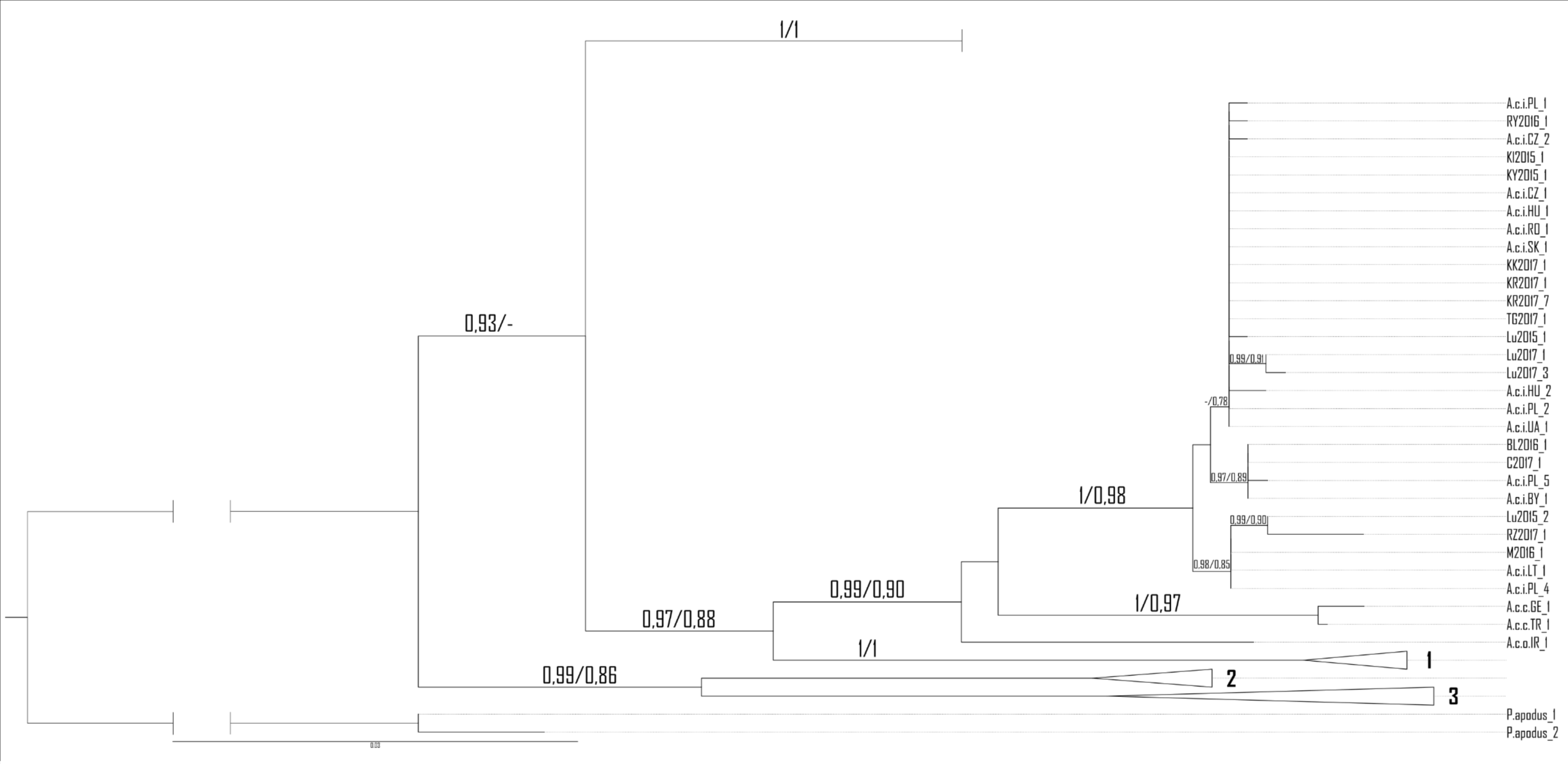

Supplement: Supplemental Information 1 — Sequences collected during this study restricted to one represent of eachND2 haplotype in the population. GenBank sample codes as in Table S3A. Numbers above branches indicate the results of the Shimodaira-Hasegawa approximate ratio test of support for branches measured (SH-aLRT), followed by Bayesian posterior probability value. “-“ represents no support for a branch. Figure_S1A –A. colchica samples, Figure_S1B –A. fragilis samples. Collapsed tree –GenBank samples: 1 –A. graeca, 2 –A. cephallonica, 3 –A. veronensis [file peerj-13-18563-s001.png]

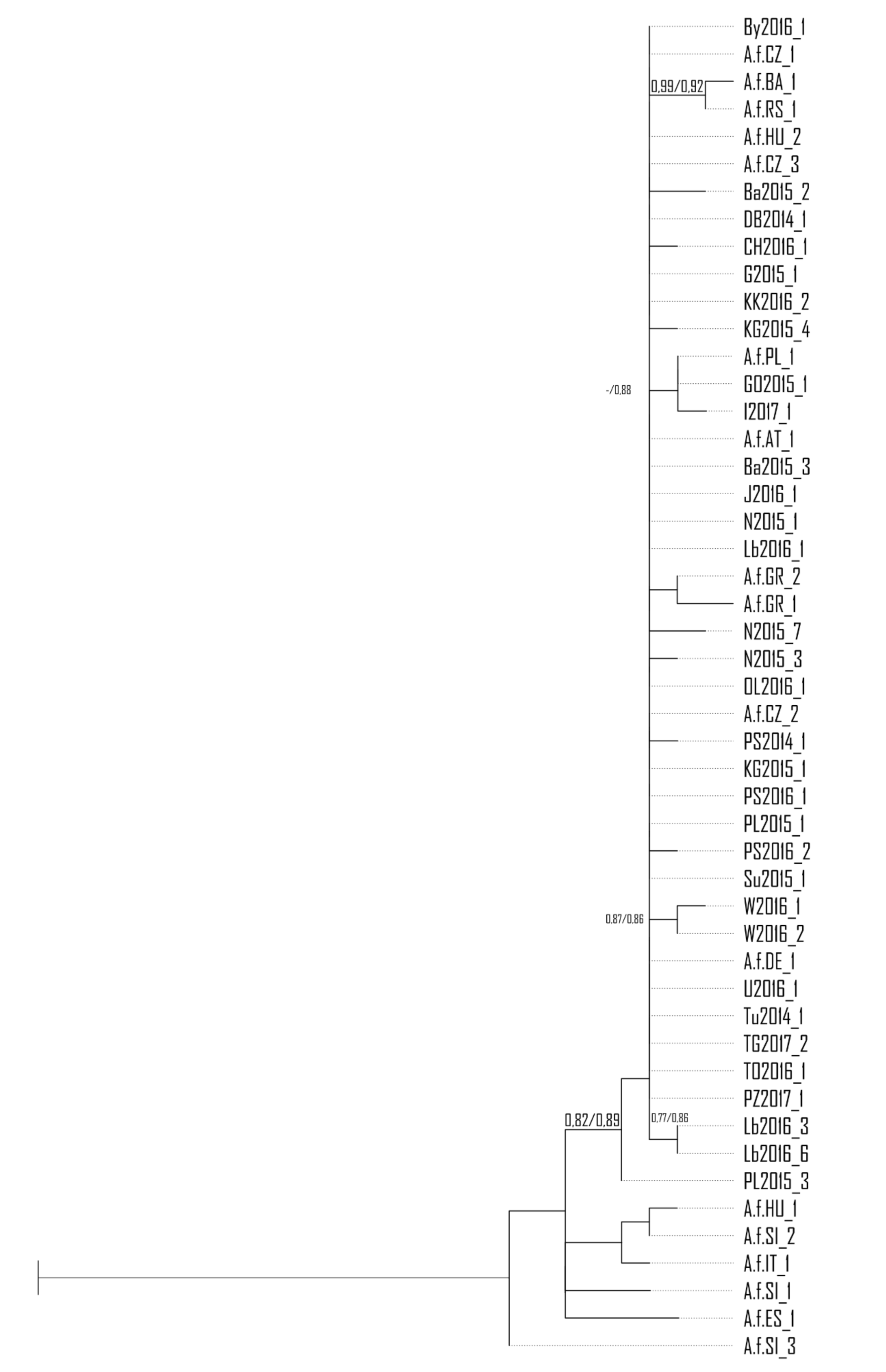

Supplement: Supplemental Information 2 — Sequences collected during this study restricted to one represent of eachND2 haplotype in the population. GenBank sample codes as in Table S3A. Numbers above branches indicate the results of the Shimodaira-Hasegawa approximate ratio test of support for branches measured (SH-aLRT), followed by Bayesian posterior probability value. “-“ represents no support for a branch. Figure_S1A –A. colchica samples, Figure_S1B –A. fragilis samples. Collapsed tree –GenBank samples: 1 –A. graeca, 2 –A. cephallonica, 3 –A. veronensis [file peerj-13-18563-s002.png]
