# Supplementary material for: Contact zone of slow worms Anguis fragilis Linnaeus, 1758 and Anguis colchica (Nordmann, 1840) in Poland
Source: PeerJ. 2025 Jan 6;13:e18563. doi: 10.7717/peerj.18563 (PMC11716018; doi:10.7717/peerj.18563)
Supplement: Supplemental Information 3 — Note: all bilateral characters counted on the right side of the head [file peerj-13-18563-s003.docx]

**Table S1A. List of the analysed morphological characters with their definitions.**

Note: all bilateral characters counted on the right side of the head

| **Character analysed** | **Definition** |
| --- | --- |
| **Snout-vent length (SVL)** | longitudinal length from the rostrum to the posterior margin of the anal scales |
| **Head dimension:** |  |
| Head height 1 (HH1) | head height in the most highest point |
| Head height 2 (HH2) | head height in the most highest point from edge of mandible |
| Head length 1 (HL1) | longitudinal length from the rostrum to the posterior margin of the occipital scale |
| Head length 2 (HL2) | longitudinal length from the rostrum to the edge of mandible |
| Head length 3 (HL3) | longitudinal length from anterior edge of orbital to the rostrum |
| Nasal to orbital length (Or-N) | longitudinal length from the anterior edge of orbital to the exterior edge of nasal opening |
| Head width (HW) | head width at the withiest point |
| Nasal opening length (NO) | horizontal length of the nasal opening |
| Length of the frontal shield (FL) | length of the frontal shield from the most external edge |
| Width of the frontal shield (FW) | width of the frontal shield from the most external edge |
| **Scalation:** |  |
| Scales round the body (SRC) | number of scales round in the body at the level of half of SVL |
| Ventral scales (V) | number of scales in a row on the ventral side of the body |
| Supralabial scales (SL) | number of supralabial scales |
| Ifralabial scales (IL) | number of infralabial scales |
| **Prefrontal scales position (P)** | A – in broad contact; B- in point contact; C – no contact, D – other pattern (Dely, 1972) |
| **Ear opening (EO)** | 0 – no visible ear opening on any side; 1 – ear opening clearly visible on the right side; 2 – ear opening clearly visible on the left side; 3 – ear opening clearly visible on the both sides |
| **Coloration:** |  |
| Dorsal spot (DP) | presence of any kind of spots on dorsal part of body |
| Dorsal line (CL) | gradient from absence to presence of dorsal line: |
| Dorsal/lateral border coloration (CT) | gradient from prominent border to no border between the dorsal and lateral coloration |
| Abdominal coloration (CV) | coloration of ventral part of the body from black to light (or other) |
| Spots after head (HP) | Gradient from presence to absence of spots behind head:  1- presence of green spots; 2 – absence of green spots; 3 – spots of a different color, |
