# Supplementary material for: Contact zone of slow worms Anguis fragilis Linnaeus, 1758 and Anguis colchica (Nordmann, 1840) in Poland
Source: PeerJ. 2025 Jan 6;13:e18563. doi: 10.7717/peerj.18563 (PMC11716018; doi:10.7717/peerj.18563)
Supplement: Supplemental Information 4 — Distances between A. colchica and A. fragilis are bolded. 27 - Celestynów (population 27); 29 - Kędzierzyn-Koźle (population 29); 40 - Tarnowskie Góry (population 40) [file peerj-13-18563-s004.docx]

| Marker | Distance p (%) | *A. colchica* | *A. cephallonica* | *A. fragilis* | *A. graeca* | *A. veronensis* | *P. apodus* |
| --- | --- | --- | --- | --- | --- | --- | --- |
| *ND2* | *A. colchica* |  |  |  |  |  |  |
|  | *A. cephallonica* | 7.78 |  |  |  |  |  |
|  | *A. fragilis* | **6.9** | 8.91 |  |  |  |  |
|  | *A. graeca* | 5.7 | 8.18 | 8.16 |  |  |  |
|  | *A. veronensis* | 9.01 | 5.99 | 9.28 | 8.67 |  |  |
|  | *P. apodus* | 12.15 | 11.83 | 13.29 | 12.24 | 12.09 |  |
|  |  | *A. colchica* | *A. fragilis* | 27 | 29 | 40 |  |
| *RAG 1* | *A. colchica* |  |  |  |  |  |  |
|  | *A. fragilis* | **0.245** |  |  |  |  |  |
|  | 27 | 0.32 | 0.17 |  |  |  |  |
|  | 29 | 0.25 | 0.018 | 0.165 |  |  |  |
|  | 40 | 0.25 | 0.018 | 0.165 | 0 |  |  |
