# Supplementary material for: Contact zone of slow worms Anguis fragilis Linnaeus, 1758 and Anguis colchica (Nordmann, 1840) in Poland
Source: PeerJ. 2025 Jan 6;13:e18563. doi: 10.7717/peerj.18563 (PMC11716018; doi:10.7717/peerj.18563)
Supplement: Supplemental Information 5 — Characters codding as in Table S1A [file peerj-13-18563-s005.docx]

| **Characters** | **Value** | **Image** |
| --- | --- | --- |
| Dorsal spot polymorphism  (DP) | DP_1_ – presence of blue spots  (Kaczmarek, 2015) | **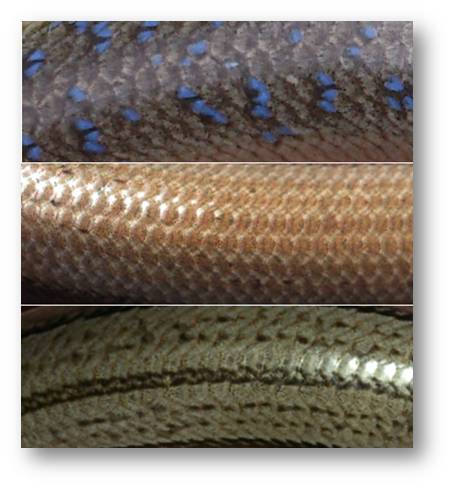** |
|  | DP_2_ – presence of green spots  (Kaczmarek, 2015) | 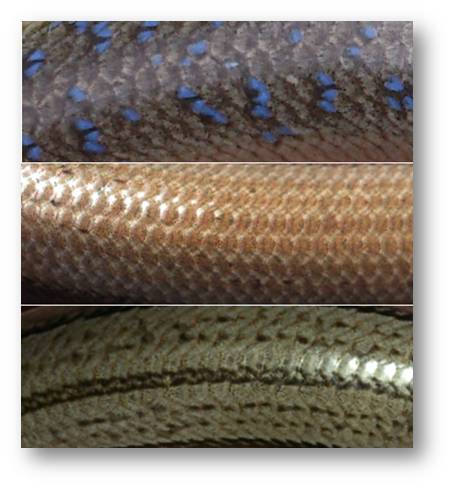 |
|  | DP_3_ - no dorsal spots | 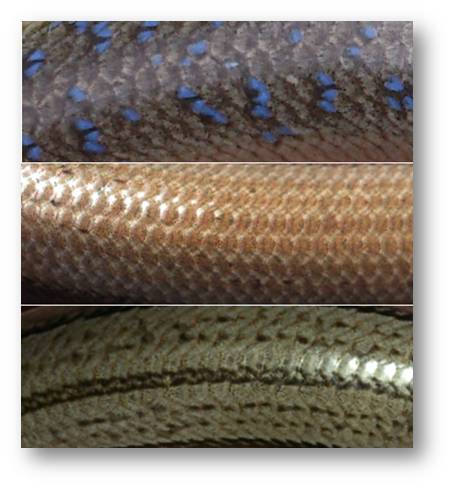 |
|  | DP_4_ – 4 lines of spots so called „pattern”  (Kaczmarek, 2015) | 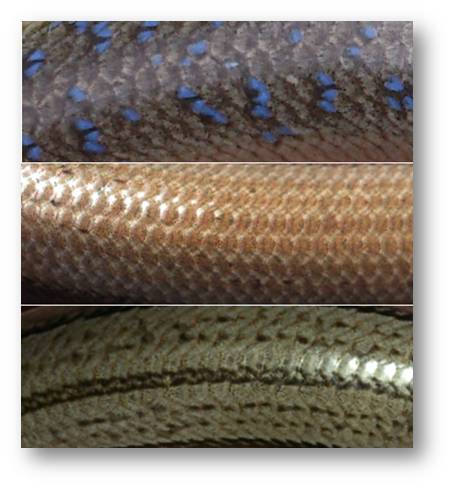 |
| Dorsal line (CL) and dorsa/lateral border (CT) | CL_1_ – No dorsal line  CT_1_ – lacking of dorsa/lateral border,  (Kaczmarek, 2015) | 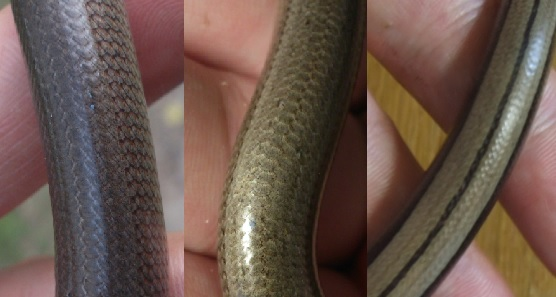 |
|  | CL_2_ – Vanishing dorsal line  CT_2_ – intermediate state of dorsa/lateral border,  (Kaczmarek, 2015) | 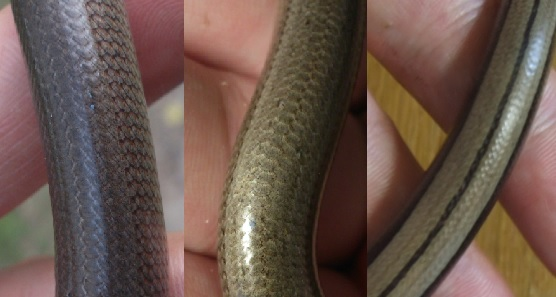 |
|  | CL_3_ – presence of dorsal line  CT_3_ – strong dorsa/lateral border,  (Kaczmarek, 2015) | 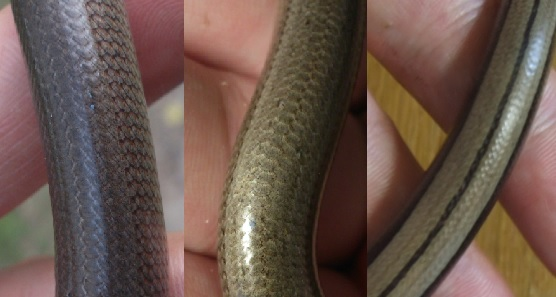 |
| Abdominal coloration (CV) | CV_1_ – Black coloration of ventral part of body  (Kaczmarek, 2015) | 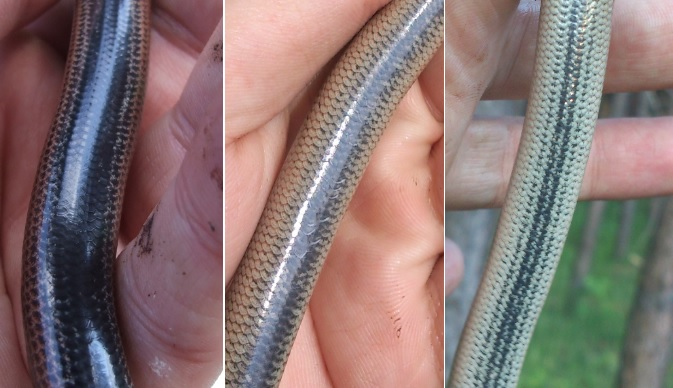 |
|  | CV_2_ – intermediate state of ventral part of body  (Kaczmarek, 2015) | 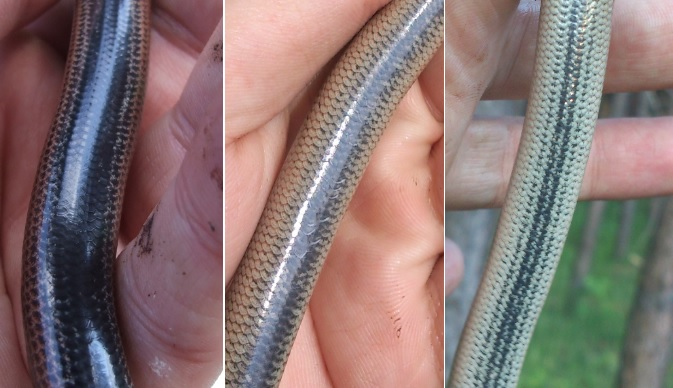 |
|  | CV_3_ – light state of ventral part of body  (Kaczmarek, 2015) | 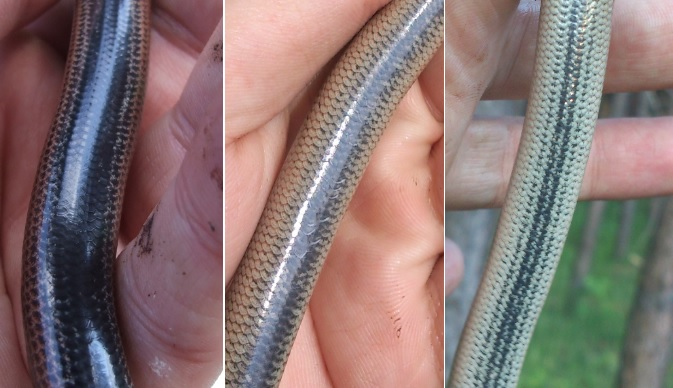 |
|  | CV_4_ – Different coloration of ventral part of body (eg. blue) (Kaczmarek et al., 2016) | 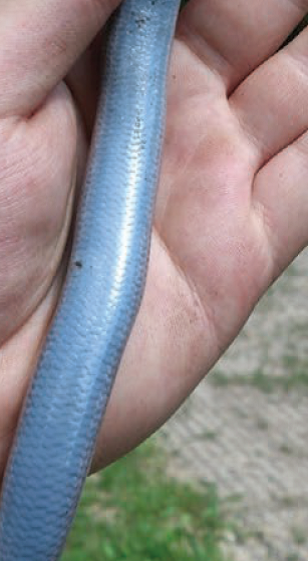 |
| Spots after head (HP) | HP_1_ – no brown spots after head  (Kaczmarek, 2015) | 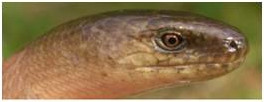 |
|  | HP_2_ – Brown spots presence after head.  HP_3_ – Spots after head with other color (no image).  (Kaczmarek, 2015) | 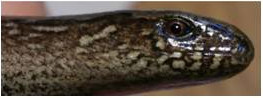 |
| Ear opening (EO) | EO_1_ – Ear opening clearly visible on both side  EO_1*_ - Ear opening clearly visible on one side  (Skórzewski, 2017) | 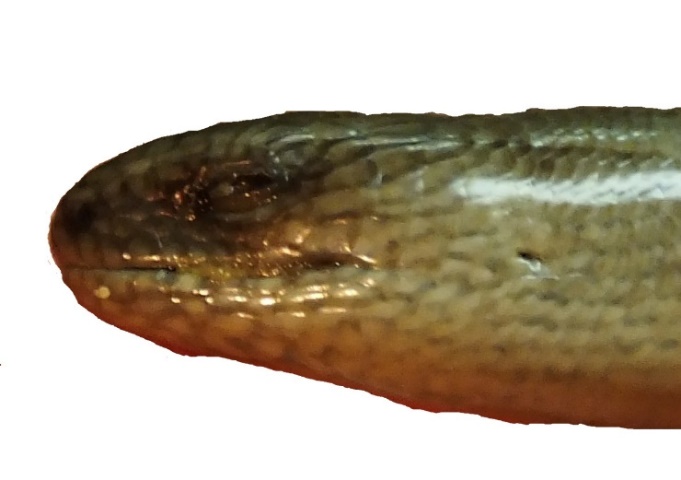 |
|  | EO_2_ – no visible ear opening  (Skórzewski, 2017) | 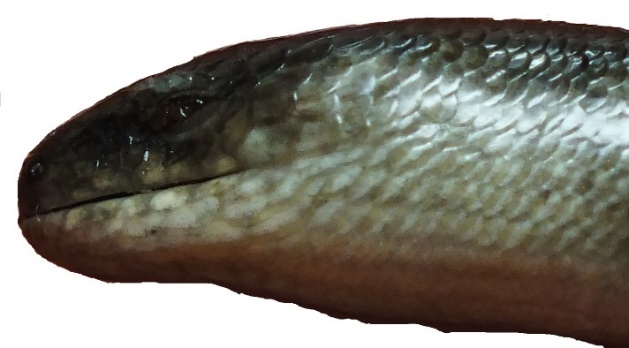 |
|  | EO_3_ – Shallow on one or both side of head,  (Skórzewski, 2017) | 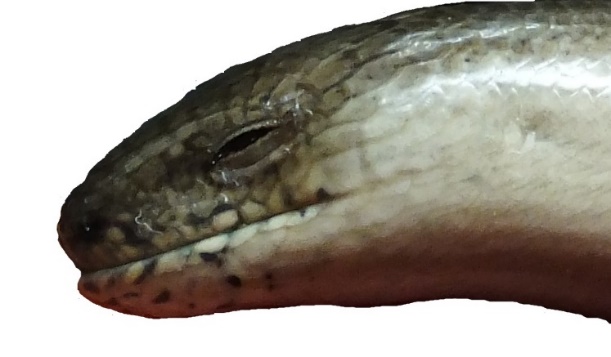 |
