# Supplementary material for: Contact zone of slow worms Anguis fragilis Linnaeus, 1758 and Anguis colchica (Nordmann, 1840) in Poland
Source: PeerJ. 2025 Jan 6;13:e18563. doi: 10.7717/peerj.18563 (PMC11716018; doi:10.7717/peerj.18563)
Supplement: Supplemental Information 6 — Characters description in Table S1A [file peerj-13-18563-s006.docx]

| **Males** |  |  | **SVL** | **HH1** | **HH2** | **HL1** | **HL2** | **HL3** | **OR_N** | **HW** | **FW** | **FL** | **NO** |
| --- | --- | --- | --- | --- | --- | --- | --- | --- | --- | --- | --- | --- | --- |
|  | **SVL** | Pearson correlation coefficient | 1 | -0.015 | -0.019 | -0.002 | 0.018 | 0.013 | -0.009 | -0.007 | -0.014 | -0.015 | -0.009 |
|  |  | Sig (both sides) |  | 0.86 | 0.83 | 0.984 | 0.834 | 0.879 | 0.922 | 0.934 | 0.87 | 0.866 | 0.917 |
|  |  | N | 132 | 132 | 132 | 132 | 132 | 132 | 132 | 132 | 132 | 132 | 132 |
| **Females** | **SVL** | Pearson correlation coefficient | 1 | 0.007 | 0.008 | 0.009 | 0.006 | -0.015 | -0.003 | 0.031 | 0.014 | 0.041 | -0.019 |
|  |  | Sig (both sides) |  | 0.945 | 0.934 | 0.924 | 0.951 | 0.883 | 0.975 | 0.752 | 0.884 | 0.681 | 0.85 |
|  |  | N | 105 | 105 | 105 | 105 | 105 | 105 | 105 | 105 | 105 | 105 | 105 |
