# Supplementary material for: Contact zone of slow worms Anguis fragilis Linnaeus, 1758 and Anguis colchica (Nordmann, 1840) in Poland
Source: PeerJ. 2025 Jan 6;13:e18563. doi: 10.7717/peerj.18563 (PMC11716018; doi:10.7717/peerj.18563)
Supplement: Supplemental Information 7 — Code - code used in the study. [file peerj-13-18563-s007.docx]

| **Code** | **Origin** | **Coordinates:** | | **GenBank Accession number** | **Source** |
| --- | --- | --- | --- | --- | --- |
|  |  | **N** | **E** |  |  |
| *A. c. colchica* GE 1. | Georgia | 41.37, 43.27 | | FJ666584 | Gvoždík et al., 2010 |
| *A. c. colchica* TR 1. | Republic of Türkiye | 41.40, 41.44 | | FJ666585.1 | Gvoždík et al., 2010 |
| *A. c. incerta* BY1 | Republic of Belarus | 52.884, 23.996 | | MF817488 | Jablonski et al., 2017 |
| *A. c. incerta* CZ 1 | Czech Republic | 49.05, 17.88 | | FJ666577.1 | Gvoždík et al., 2010 |
| *A. c. incerta* CZ 2 | Czech Republic | 49.58, 18.10 | | FJ666578.1 | Gvoždík et al., 2010 |
| *A. c. incerta* HU 1 | Hungary | 48.02, 18.91 | | KF736829 | Szabó & Vörös, 2014 |
| *A. c. incerta* HU 2 | Hungary | 47.82, 19.97 | | KF736830 | Szabó & Vörös, 2014 |
| *A. c. incerta* LT 1 | Republic of Lithuania | 55.33, 26.10 | | FJ666581.1 | Gvoždík et al., 2010 |
| *A. c. incerta* PL 1 | Republic od Poland | 52.65, 23.05 | | FJ666579.1 | Gvoždík et al., 2010 |
| *A. c. incerta* PL 2 | Republic od Poland | 49.235,22.557 | | MF817484 | Jablonski et al., 2017 |
| *A. c. incerta* PL 3 | Republic od Poland | 49.686, 21.787 | | MF817487 | Jablonski et al., 2017 |
| *A. c. incerta* PL 4 | Republic od Poland | 51.324, 21.958 | | MF817492 | Jablonski et al., 2017 |
| *A. c. incerta* PL 5 | Republic od Poland | 50.507, 22.791 | | MF817483 | Jablonski et al., 2017 |
| *A. c. incerta* PL 6 | Republic od Poland | 50.226, 18.125 | | MF817491 | Jablonski et al., 2017 |
| *A. c. incerta* PL 7 | Republic od Poland | 50.097, 18.260 | | MF817489 | Jablonski et al., 2017 |
| *A. c. incerta* RO1 | Romania | 46.83, 23.62 | | FJ666580.1 | Gvoždík et al., 2010 |
| *A. c. incerta* SK 1 | Republic od Slovakia | 48.92, 18.95 | | FJ666576.1 | Gvoždík et al., 2010 |
| *A. c. incerta* UA 1 | Ukraine | 49.206, 22.736 | | MF817486.1 | Jablonski et al., 2017 |
| *A. c. orientalis* IR 2 | Islamic Republic of Iran | 36.65, 51.50 | | FJ666583.1 | Gvoždík et al., 2010 |
| *A. c. orientalis* IR1 | Islamic Republic of Iran | 38.20, 48.87 | | FJ666582.1 | Gvoždík et al., 2010 |
| *A. cepchallonica* GR 1 | Hellenic Republic | 37.31, 22.15 | | KJ634792 | Thanou et al., 2014 |
| *A. cepchallonica* GR 2 | Hellenic Republic | 37.15, 22.19 | | KJ634793 | Thanou et al., 2014 |
| *A. cepchallonica* GR 3 | Hellenic Republic | 37.86, 21.89 | | KJ634789 | Thanou et al., 2014 |
| *A. cepchallonica* GR 4 | Hellenic Republic | 37.21, 21.73 | | KJ634785 | Thanou et al., 2014 |
| *A. cepchallonica* GR 5 | Hellenic Republic | 37.68, 22.11 | | KJ634787 | Thanou et al., 2014 |
| *A. cepchallonica* GR 6 | Hellenic Republic | 37.63, 22.15 | | KJ634786 | Thanou et al., 2014 |
| *A. cepchallonica* GR 7 | Hellenic Republic | 38.19, 20.67 | | KJ634790 | Thanou et al., 2014 |
| *A. cepchallonica* GR 8 | Hellenic Republic | 38.27, 21.95 | | KJ634784 | Thanou et al., 2014 |
| *A. cepchallonica* GR 9 | Hellenic Republic | 37.67, 22.02 | | KJ634788 | Thanou et al., 2014 |
| *A. fragilis* AT 1 | Republic of Austria | 47.55, 13.66 | | KC881543.1 | Gvoždík et al., 2013 |
| *A. fragilis* BA 1 | Bosnia and Herzegovina | 43.03, 18.49 | | KC881542.1 | Gvoždík et al., 2013 |
| *A. fragilis* CZ 1 | Czech Republic | 50.33, 13.10 | | FJ666554.1 | Gvoždík et al., 2010 |
| *A. fragilis* CZ 2 | Czech Republic | 49.41, 15.52 | | FJ666555 | Gvoždík et al., 2010 |
| *A. fragilis* CZ 3 | Czech Republic | 50.25, 17.35 | | FJ666556.1 | Gvoždík et al., 2010 |
| *A. fragilis* DE 1 | Federal Republic of Germany | 48.17, 11.47 | | KC881544.1 | Gvoždík et al., 2013 |
| *A. fragilis* ES 1 | Kingdom of Spain | 43.36, −4.86 | | KF736837.1 | Szabó & Vörös, 2014 |
| *A. fragilis* GR1 | Hellenic Republic | 40.89, 24.06 | | FJ666557.1 | Gvoždík et al., 2010 |
| *A. fragilis* GR2 | Hellenic Republic | 41.37, 24.63 | | FJ666558.1 | Gvoždík et al., 2010 |
| *A. fragilis* HU 1 | Hungary | 47.64, 18.95 | | KF736835.1 | Szabó & Vörös, 2014 |
| *A. fragilis* HU 2 | Hungary | 47.53, 18.99 | | KF736831.1 | Szabó & Vörös, 2014 |
| *A. fragilis* IT 1 | Italian Republic | 46.40, 13.17 | | KC881539.1 | Gvoždík et al., 2013 |
| *A. fragilis* PL 1 | Republic od Poland | 53.613, 19.529 | | MF817472 | Jablonski et al., 2017 |
| *A. fragilis* PL 2 | Republic od Poland | 51.528, 17.341 | | MF817476 | Jablonski et al., 2017 |
| *A. fragilis* PL 3 | Republic od Poland | 50.897, 16.730 | | MF817475 | Jablonski et al., 2017 |
| *A. fragilis* PL 4 | Republic od Poland | 52.053, 21.041 | | MF817468 | Jablonski et al., 2017 |
| *A. fragilis* PL 5 | Republic od Poland | 51.629, 15.061 | | MF817461.1 | Jablonski et al., 2017 |
| *A. fragilis* PL 6 | Republic od Poland | 51.731, 18.821 | | MF817469.1 | Jablonski et al., 2017 |
| *A. fragilis* RS 1 | Republic of Serbia | 43.86, 19.84 | | KC881541.1 | Gvoždík et al., 2013 |
| *A. fragilis* SI 1 | Republic of Slovenia | 45.52, 13.58 | | KF736836.1 | Szabó & Vörös, 2014 |
| *A. fragilis* SI 2 | Republic of Slovenia | 46.29, 13.90 | | FJ666559.1 | Gvoždík et al., 2010 |
| *A. fragilis* SI 3 | Republic of Slovenia | 45.60, 13.95 | | KC881540.1 | Gvoždík et al., 2013 |
| *A. graeca* AL 1 | Republic of Albania | 40.32, 20.67 | | FJ666563 | Gvoždík et al., 2010 |
| *A. graeca* AL 2 | Republic of Albania | 40.32, 20.67 | | FJ666564 | Gvoždík et al., 2010 |
| *A. graeca* AL 3 | Republic of Albania | 40.68, 19.66 | | FJ666566 | Gvoždík et al., 2010 |
| *A. graeca* GR 1 | Hellenic Republic | 38.49, 22.06 | | FJ666560 | Gvoždík et al., 2010 |
| *A. graeca* GR 2 | Hellenic Republic | 39.54, 21.47 | | FJ666561 | Gvoždík et al., 2010 |
| *A. graeca* GR 3 | Hellenic Republic | 39.89, 22.62 | | FJ666562 | Gvoždík et al., 2010 |
| *A. graeca* GR 4 | Hellenic Republic | 40.05, 20.76 | | FJ666565 | Gvoždík et al., 2010 |
| *A. graeca* RS 1 | Republic of Serbia | 43.86, 19.84 | | FJ666567 | Gvoždík et al., 2010 |
| *A. veronensis* FR 1 | French Republic | 44.07, 07.51 | | KC881552 | Gvoždík et al., 2013 |
| *A. veronensis* IT 1 | Italian Republic | 40.31, 15.58 | | KC881548 | Gvoždík et al., 2013 |
| *A. veronensis* IT 2 | Italian Republic | 40.04, 16.10 | | KC881549 | Gvoždík et al., 2013 |
| *A. veronensis* IT 3 | Italian Republic | 44.31, 09.20 | | KC881550 | Gvoždík et al., 2013 |
| *A. veronensis* IT 4 | Italian Republic | 44.20, 08.37 | | KC881551 | Gvoždík et al., 2013 |
| *A. veronensis* IT 5 | Italian Republic | 45.75, 09.94 | | KC881553 | Gvoždík et al., 2013 |
| *A. veronensis* IT 6 | Italian Republic | 43.47, 12.63 | | KC881554 | Gvoždík et al., 2013 |
| *A. veronensis* IT 7 | Italian Republic | 43.38, 12.11 | | KC881555 | Gvoždík et al., 2013 |
| *A. veronensis* IT 8 | Italian Republic | 46.35, 12.14 | | KC881561 | Gvoždík et al., 2013 |
| *P. apodus* 1 | State of Israel | 33.04, 35,22 | | MF547724 | Jandzik et al., 2018 |
| *P. apodus* 2 | Republic of Türkiye | 36.20, 36.18 | | MF547723 | Jandzik et al., 2018 |
