# Supplementary material for: Contact zone of slow worms Anguis fragilis Linnaeus, 1758 and Anguis colchica (Nordmann, 1840) in Poland
Source: PeerJ. 2025 Jan 6;13:e18563. doi: 10.7717/peerj.18563 (PMC11716018; doi:10.7717/peerj.18563)
Supplement: Supplemental Information 8 — Characters described in Table S1A [file peerj-13-18563-s008.docx]

| **Males** | | | | **Females** | | |
| --- | --- | --- | --- | --- | --- | --- |
|  | **PC** | | | **PC** | | |
|  | **1** | **2** | **3** | **1** | **2** | **3** |
| Eigenvalues: | 6.152 | 0.981 | 0.709 | 5.484 | 1.009 | 0.753 |
| % Variance | 61.523 | 9.81 | 7.091 | 54.843 | 10.085 | 7.532 |
| **Character** | | | | | | |
| HH1 | 0.847 | -0.337 | 0.145 | 0.782 | -0.351 | -0.214 |
| HH2 | 0.817 | -0.27 | 0.081 | 0.772 | -0.377 | 0.129 |
| HL1 | 0.786 | 0.385 | -0.217 | 0.828 | 0.304 | -0.111 |
| HL2 | 0.813 | 0.048 | 0.02 | 0.764 | -0.158 | -0.304 |
| HL3 | 0.799 | 0.18 | -0.333 | 0.744 | 0.361 | -0.069 |
| OR-N | 0.78 | 0.075 | -0.441 | 0.756 | 0.159 | 0.087 |
| HW | 0.807 | -0.398 | 0.05 | 0.746 | -0.239 | -0.29 |
| FW | 0.746 | 0.103 | 0.455 | 0.661 | 0.097 | 0.585 |
| FL | 0.61 | 0.626 | 0.344 | 0.608 | 0.578 | -0.1 |
| NO | 0.815 | -0.213 | -0.018 | 0.721 | -0.264 | 0.371 |
