# Supplementary material for: Contact zone of slow worms Anguis fragilis Linnaeus, 1758 and Anguis colchica (Nordmann, 1840) in Poland
Source: PeerJ. 2025 Jan 6;13:e18563. doi: 10.7717/peerj.18563 (PMC11716018; doi:10.7717/peerj.18563)
Supplement: Supplemental Information 9 — Code - code used in the study. [file peerj-13-18563-s009.docx]

| **Code** | **Origin** | **Coordinates** | **Haplotype** | **GenBank Accession number** | **Source** |
| --- | --- | --- | --- | --- | --- |
| Ophi_1 | Museum voucher number MVZ-RM10468A | - | - | AY662602 | Townsend et al., 2004 |
