# Supplementary material for: Contact zone of slow worms Anguis fragilis Linnaeus, 1758 and Anguis colchica (Nordmann, 1840) in Poland
Source: PeerJ. 2025 Jan 6;13:e18563. doi: 10.7717/peerj.18563 (PMC11716018; doi:10.7717/peerj.18563)
Supplement: Supplemental Information 10 — G - compared groups; PC - principal components; Statistical significance is bolded [file peerj-13-18563-s010.docx]

|  | **Males** | | | **Females** | | |
| --- | --- | --- | --- | --- | --- | --- |
| **PC** | **G1** | **G2** | **Sig.** | **G1** | **G2** | **Sig.** |
| **1** | *A. fragilis* | *A. colchica* | 0.786 | *A. fragilis* | *A. colchica* | **0.001** |
|  | *A. fragilis* | Grey zone | 0.724 | *A. fragilis* | Grey zone | 0.988 |
|  | *A. colchica* | Grey zone | 0.308 | *A. colchica* | Grey zone | **0.034** |
| **2** | *A. fragilis* | *A. colchica* | **0.001** | *A. fragilis* | *A. colchica* | **0.001** |
|  | *A. fragilis* | Grey zone | 0.429 | *A. fragilis* | Grey zone | 0.63 |
|  | *A. colchica* | Grey zone | **0.001** | *A. colchica* | Grey zone | **0.004** |
| **3** | *A. fragilis* | *A. colchica* | 0.971 | *A. fragilis* | *A. colchica* | **0.005** |
|  | *A. fragilis* | Grey zone | 0.997 | *A. fragilis* | Grey zone | 0.186 |
|  | *A. colchica* | Grey zone | 0.966 | *A. colchica* | Grey zone | 0.923 |
