# Supplementary material for: Contact zone of slow worms Anguis fragilis Linnaeus, 1758 and Anguis colchica (Nordmann, 1840) in Poland
Source: PeerJ. 2025 Jan 6;13:e18563. doi: 10.7717/peerj.18563 (PMC11716018; doi:10.7717/peerj.18563)
Supplement: Supplemental Information 12 — Characters description in Table S1A [file peerj-13-18563-s012.docx]

| **Sexes** | **Function** | **Eigenvalues:** | **% of Variance** | **Canonical correlation** |
| --- | --- | --- | --- | --- |
| Males | 1 | 0.534 | 95.3 | 0.590 |
|  | 2 | 0.027 | 4.7 | 0.161 |
| Females | 1 | 0.604 | 82.7 | 0.614 |
|  | 2 | 0.126 | 17.3 | 0.335 |
