# Supplementary material for: Contact zone of slow worms Anguis fragilis Linnaeus, 1758 and Anguis colchica (Nordmann, 1840) in Poland
Source: PeerJ. 2025 Jan 6;13:e18563. doi: 10.7717/peerj.18563 (PMC11716018; doi:10.7717/peerj.18563)
Supplement: Supplemental Information 13 — Significant results in bold. [file peerj-13-18563-s013.docx]

| **Sexes** | **Test of functions** | **Wilk's Lambda** | **χ2** | **df** | **Sig.** |
| --- | --- | --- | --- | --- | --- |
| Males | 1 through 2 | 0.635 | 55.527 | 20 | **0.001** |
|  | 2 | 0.974 | 3.267 | 9 | 0.953 |
| Females | 1 through 2 | 0.554 | 57.654 | 20 | **0.001** |
|  | 2 | 0.888 | 11.601 | 9 | 0.237 |
