# Supplementary material for: Contact zone of slow worms Anguis fragilis Linnaeus, 1758 and Anguis colchica (Nordmann, 1840) in Poland
Source: PeerJ. 2025 Jan 6;13:e18563. doi: 10.7717/peerj.18563 (PMC11716018; doi:10.7717/peerj.18563)
Supplement: Supplemental Information 14 — Characters of the highest within-group correlations with each function are bolded. Characters description in Table S1A [file peerj-13-18563-s014.docx]

|  | **Males** | |  | **Females** | |
| --- | --- | --- | --- | --- | --- |
|  | **CV** | |  | **CV** | |
| **Character** | 1 | 2 | **Character** | 1 | 2 |
| FL | **0.503** | 0.238 | FL | **0.677** | 0.305 |
| HL1 | **0.488** | 0.392 | HL1 | **0.675** | 0.069 |
| HL3 | 0.218 | 0.057 | HL3 | **0.589** | -0.13 |
| HW | **-0.178** | **0.646** | HL2 | **0.398** | -0.174 |
| HH1 | -0.077 | **0.550** | HW | **0.279** | 0.206 |
| OR_N | 0.031 | **0.421** | HH1 | **0.269** | -0.174 |
| HH2 | -0.134 | **0.329** | OR_N | **0.398** | 0.325 |
| FW | 0.054 | **0.305** | FW | 0.054 | **0.402** |
| HL2 | 0.234 | **0.263** | NO | 0.189 | **0.233** |
| NO | -0.061 | **0.219** | HH2 | 0.113 | **-0.211** |
