# Supplementary material for: Contact zone of slow worms Anguis fragilis Linnaeus, 1758 and Anguis colchica (Nordmann, 1840) in Poland
Source: PeerJ. 2025 Jan 6;13:e18563. doi: 10.7717/peerj.18563 (PMC11716018; doi:10.7717/peerj.18563)
Supplement: Supplemental Information 15 — Significant results in bold. Characters description in Table S1A. GZ - grey zone specimens. [file peerj-13-18563-s015.docx]

| **Males** | | | | | | | | | | | |
| --- | --- | --- | --- | --- | --- | --- | --- | --- | --- | --- | --- |
| **Character** | **Means and SD** | | | | |  | **Wilks's lambda** | **F** | **df1** | **df2** | **sig.** |
|  | *A. fragilis* | SD | *A. colchica* | SD | GZ | SD |  |  |  |  |  |
| HH1 | 7.20 | 0.95 | 7.07 | 0.63 | 7.09 | 0.77 | 0.994 | 0.364 | 2 | 129 | 0.696 |
| HH2 | 4.08 | 0.55 | 3.95 | 0.38 | 3.98 | 0.57 | 0.986 | 0.921 | 2 | 129 | 0.401 |
| HL1 | 13.74 | 0.92 | 14.37 | 0.54 | 13.62 | 1.10 | 0.884 | 8.465 | 2 | 129 | **<0.001** |
| HL2 | 9.07 | 1.21 | 9.45 | 0.81 | 8.96 | 1.30 | 0.97 | 1.999 | 2 | 129 | 0.14 |
| HL3 | 4.88 | 0.43 | 5.01 | 0.31 | 4.85 | 0.47 | 0.974 | 1.716 | 2 | 129 | 0.184 |
| OR_N | 3.55 | 0.39 | 3.54 | 0.28 | 3.45 | 0.38 | 0.989 | 0.75 | 2 | 129 | 0.475 |
| HW | 9.53 | 1.22 | 9.22 | 0.78 | 9.49 | 1.01 | 0.983 | 1.099 | 2 | 129 | 0.336 |
| FW | 3.68 | 0.47 | 3.69 | 0.37 | 3.58 | 0.44 | 0.99 | 0.62 | 2 | 129 | 0.539 |
| FL | 4.22 | 0.43 | 4.53 | 0.29 | 4.19 | 0.51 | 0.88 | 8.792 | 2 | 129 | **<0.001** |
| NO | 2.92 | 0.39 | 2.87 | 0.36 | 2.87 | 0.38 | 0.995 | 0.313 | 2 | 129 | 0.732 |
| **Females** | | | | | | | | | | | |
| HH1 | 6.10 | 0.66 | 6.42 | 0.69 | 6.26 | 0.79 | 0.955 | 2.418 | 2 | 102 | 0.094 |
| HH2 | 3.40 | 0.47 | 3.49 | 0.40 | 3.51 | 0.37 | 0.987 | 0.682 | 2 | 102 | 0.508 |
| HL1 | 12.26 | 0.72 | 13.00 | 0.61 | 12.29 | 0.76 | 0.784 | 14.026 | 2 | 102 | **<0.001** |
| HL2 | 7.71 | 0.86 | 8.23 | 0.69 | 7.91 | 0.72 | 0.91 | 5.074 | 2 | 102 | **0.008** |
| HL3 | 4.32 | 0.40 | 4.68 | 0.34 | 4.37 | 0.42 | 0.827 | 10.674 | 2 | 102 | **<0.001** |
| OR_N | 3.14 | 0.28 | 3.30 | 0.23 | 3.06 | 0.34 | 0.902 | 5.554 | 2 | 102 | **0.005** |
| HW | 7.99 | 0.83 | 8.33 | 0.77 | 7.85 | 0.90 | 0.95 | 2.677 | 2 | 102 | 0.074 |
| FW | 3.24 | 0.39 | 3.24 | 0.32 | 3.08 | 0.39 | 0.978 | 1.133 | 2 | 102 | 0.326 |
| FL | 3.72 | 0.36 | 4.07 | 0.32 | 3.65 | 0.26 | 0.776 | 14.716 | 2 | 102 | **<0.001** |
| NO | 2.48 | 0.31 | 2.55 | 0.21 | 2.42 | 0.29 | 0.972 | 1.445 | 2 | 102 | 0.24 |
