# Supplementary material for: Contact zone of slow worms Anguis fragilis Linnaeus, 1758 and Anguis colchica (Nordmann, 1840) in Poland
Source: PeerJ. 2025 Jan 6;13:e18563. doi: 10.7717/peerj.18563 (PMC11716018; doi:10.7717/peerj.18563)
Supplement: Supplemental Information 16 — Statistically significant results are bolded. Asterisk indicates results obtained with MCMC methods. Characters’ codes as in Tables S1A, S2A [file peerj-13-18563-s016.docx]

| *A. fragilis* | | | | | | | *A. colchica* | | | | | **Test** | **Value** | **DF** | **Sig.** | **Test 2** | **Value** | **Sig.** |
| --- | --- | --- | --- | --- | --- | --- | --- | --- | --- | --- | --- | --- | --- | --- | --- | --- | --- | --- |
| **Character** | **Sex** | **n** | **M** | **SD** | **min** | **max** | **n** | **M** | **SD** | **min** | **max** |  |  |  |  |  |  |  |
| SVL | ♂ | 64 | 168.06 | 22.89 | 122 | 225 | 41 | 189.13 | 27.65 | 143 | 246 | t-Student | -4.236 | 103 | **<0.001** | Levene's test | 1.167 | 0.283 |
|  | ♀ | 52 | 162.07 | 20.87 | 121 | 203 | 40 | 185.71 | 37.42 | 120 | 258 | t-Student | -3.590 | 57.383 | **0.001** | Levene's test | 19.415 | **<0.001** |
| V | ♂ | 57 | 128.86 | 5.136 | 112 | 145 | 39 | 133.74 | 5.34 | 116 | 141 | t-Student | -4.501 | 94 | **<0.001** | Levene's test | 0.213 | 0.645 |
|  | ♀ | 49 | 128.75 | 5.432 | 111 | 138 | 39 | 134.76 | 5.993 | 119 | 144 | t-Student | -4.911 | 86 | **<0.001** | Levene's test | 0.484 | 0.489 |
| SRC | ♂ | 62 | 24.82 | 0.713 | 24 | 26 | 41 | 27.88 | 0.6 | 27 | 29 | Fisher’s Exact test | 120.936 | 5 | **<0.001*** | Cramer’s V | 1 | **<0.001*** |
|  | ♀ | 51 | 24.9 | 0.834 | 24 | 26 | 40 | 27.8 | 1.01 | 26 | 31 | Fisher’s Exact test | 99.319 |  | **<0.001** | Cramer’s V | 0.979 | **<0.001*** |
| SL | ♂ | 62 | 9.05 | 0.422 | 8 | 10 | 41 | 8.95 | 0.444 | 8 | 10 | Fisher’s Exact test | 1.346 |  | 0.557* | Cramer’s V | 0.114 | 0.511 |
|  | ♀ | 50 | 9 | 0.349 | 8 | 10 | 39 | 9 | 0.455 | 8 | 10 | Fisher’s Exact test | 1.51 |  | 0.529* | Cramer’s V | 0.126 | 0.616* |
| IL | ♂ | 63 | 9.06 | 0.396 | 8 | 10 | 40 | 9.05 | 0.389 | 8 | 10 | Fisher’s Exact test | 0.176 |  | 1* | Cramer’s V | 0.018 | 1* |
|  | ♀ | 50 | 9 | 0.348 | 8 | 10 | 39 | 9 | 0.32 | 8 | 10 | Fisher’s Exact test | 0.359 |  | 1* | Cramer’s V | 0.019 | 1* |
| P |  | **n** | **Type A** | **Type B** | **Type C** | **Other** | **n** | **Type A** | **Type B** | **Type C** | **Other** |  |  |  |  |  |  |  |
|  | ♂ | 62 | 38 (61.3%) | 17 (27.4%) | 7 (11.3%) | 0 | 41 | 2 (4.9%) | 8 (19.5%) | 31 (75.6%) | 0 | 𝜒2 | 48.534 | 2 | **<0.001** | Cramer’s V | 0.686 | **<0.001** |
|  | ♀ | 52 | 38 (73.1%) | 8 (15.4%) | 6 (11.5%) | 0 | 40 | 1 (2.5%) | 7 (17.5%) | 32 (80%) | 0 | 𝜒2 | 52.283 | 2 | **<0.001** | Cramer’s V | 0.754 | **<0.001** |
| DP |  | **n** | **DP1** | **DP2** | **DP3** | **DP4** | **n** | **DP1** | **DP2** | **DP3** | **DP4** |  |  |  |  |  |  |  |
|  | ♂ | 51 | 11 (21.6%) | 8 (15.7%) | 26 (51%) | 6 (11.8%) | 41 | 30 (73.2%) | 3 (7.3%) | 8 (19.5%) | 0 | Fisher’s Exact test | 25.868 |  | **<0.001*** | Cramer’s V | 0.53 | **<0.001*** |
|  | ♀ | 51 | 0 | 6 (11.8%) | 32 (62.7%) | 13 (25.5%) | 38 | 7 (18.4%) | 13 (34.2%) | 14 (36.8%) | 4 (10.5%) | Fisher’s Exact test | 19.823 |  | **<0.001*** | Cramer’s V | 0.474 | **<0.001*** |
| CV |  | **n** | **CV1** | **CV2** | **CV3** | **CV4** | **n** | **CV1** | **CV2** | **CV3** | **CV4** |  |  |  |  |  |  |  |
|  | ♂ | 52 | 7 (13.5%) | 18 (34.6%) | 27 (51.9%) | 0 | 40 | 14 (35%) | 19 (47.5%) | 6 (15%) | 1 (2.5%) | 𝜒2 | 15.421 | 3 | **<0.001** | Cramer’s V | 409 | **<0.001** |
|  | ♀ | 51 | 44 (86.3%) | 4 (7.8%) | 3 (5.9%) | 0 | 38 | 29 (76.3%) | 5 (13.2%) | 1 (2.6%) | 3 (7.9%) | Fisher’s Exact test | 4.942 |  | 0.164* | Cramer’s V | 0.247 | 0.150* |
| CL |  | **n** | **CL1** | **CL2** | **CL3** |  | **n** | **CL1** | **CL2** | **CL3** |  |  |  |  |  |  |  |  |
|  | ♂ | 52 | 4 (7.7%) | 37 (71.2%) | 21 (21.2%) |  | 40 | 3 (7.5%) | 30 (75%) | 7 (17.5%) |  | Fisher’s Exact test | 0.277 |  | 0.936* | Cramer’s V | 0.047 | 0.936* |
|  | ♀ | 51 | 39 (74.5%) | 8 (15.7%) | 5 (9.8%) |  | 38 | 27 (71.1%) | 8 (21.1%) | 3 (7.9%) |  | Fisher’s Exact test | 0.543 |  | 0.771* | Cramer’s V | 0.073 | 0.771* |
| CT |  | **n** | **CT1** | **CT2** | **CT3** |  | **n** | **CT1** | **CT2** | **CT3** |  |  |  |  |  |  |  |  |
|  | ♂ | 52 | 31 (59.6 %) | 16 (30.8%) | 5 (9.6%) |  | 40 | 25 (62.5%) | 8 (20%) | 7 (17.5%) |  | 𝜒2 | 2.114 | 2 | 0.348 | Cramer’s V | 0.152 | 0.348 |
|  | ♀ | 51 | 3 (5.9%) | 3 (5.9%) | 45 (88.2%) |  | 38 | 4 (10.5%) | 2 (5.3%) | 32 (84.2%) |  | Fisher’s Exact test | 0.798 |  | 0.808* | Cramer’s V | 0.086 | 0.722 |
| HP |  | **n** | **HP1** | **HP2** | **HP3** |  | **n** | **HP1** | **HP2** | **HP3** |  |  |  |  |  |  |  |  |
|  | ♂ | 53 | 19 (35.8%) | 31 (58.5%) | 3 (5.7%) |  | 40 | 15 (37.5%) | 24 (60%) | 1 (2.5%) |  | Fisher’s Exact test | 0.524 |  | 0.876* | Cramer’s V | 0.077 | 0.772* |
|  | ♀ | 51 | 47 (92.2%) | 4 (7.8%) | 0 |  | 38 | 28 (73.7%) | 10 (26.3%) | 0 |  | 𝜒2 | 5.605 | 1 | **0.018** | Cramer’s V | 0.251 | 0.018 |
| EO |  | **n** | **EO1** | **EO1*** | **EO2** | **EO3** | **n** | **EO1** | **EO1*** | **EO2** | **EO3** |  |  |  |  |  |  |  |
|  | ♂ | 62 | 62 (100%) | 0 | 0 | 0 | 41 | 0 (0%) | 5 (12.2%) | 5 (12.2%) | 31 (75.6%) | Fisher’s Exact test | 126.055 |  | **<0.001*** | Cramer’s V | 1 | **<0.001*** |
|  | ♀ | 52 | 52 (100%) | 0 | 0 | 0 | 40 | 0 | 1 (2.5%) | 2 (5%) | 37 (92.5%) | Fisher’s Exact test | 115.871 |  | **<0.001*** | Cramer’s V | 1 | **<0.001*** |
